# Ecthyma gangrenosum: a rare manifestation of Stenotrophomonas maltophilia infection in acute myelogenous leukemia patient

**DOI:** 10.1016/j.idcr.2021.e01304

**Published:** 2021-10-07

**Authors:** Gawahir A. Ali, Wael Goravey, Muna Al Maslamani, Ali S. Omrani

**Affiliations:** Department of Infectious Diseases, Communicable Diseases Centre, Hamad Medical Corporation, Doha, Qatar

**Keywords:** Ecthyma gangrenosum, Stenotrophomonas maltophilia, AML, Trimethoprim-sulfamethoxazole, Hematological malignancy

## Abstract

Ecthyma gangrenosum is a cutaneous infection typically associated with Pseudomonas aeruginosa. However, it is rarely caused by Stenotrophomonas maltophilia which might be overlooked leading to devastating consequences. We describe this case to avoid delays in the diagnosis and treatment of this aggressive infection, especially in immunocompromised patients.

A 32-year-old female newly diagnosed with AML on cytarabine and idarubicin (7 + 3) chemotherapy regimen developed fever and a right inguinal tender skin lesion over three days duration. Examination revealed pyrexia of 38.40 C, 1–2 cm tender red papule in the right inguinal area which progressed the next day to a larger necrotic hemorrhagic vesicle that eventually ruptured (Fig. 1). Investigations showed an absolute neutrophil count of 0.00, Platelets 35,000/µL (150,000 to 450,000), CRP 233 mg/L (0–5), and procalcitonin 11 µg/L (< 0.05). She was started on meropenem 1 g q8 and was presumptively diagnosed with ecthyma gangrenosum. Amikacin 15 mg/kg/24hrs was added the day after when the fever continued. Blood cultures were negative, and the biopsy was deferred because of thrombocytopenia and profound neutropenia. Gram staining from the ruptured vesicle showed numerous gram-negative rods and the culture grew *Stenotrophomonas maltophilia*. The fever settled quickly when trimethoprim-sulfamethoxazole (TMP-SMX) and levofloxacin were added. Later, *Stenotrophomonas maltophilia* turn to be sensitive to both antimicrobials which were continued for two weeks. The lesion showed an impressive clinical response and healed completely within 3 weeks.

**Fig. 1:**
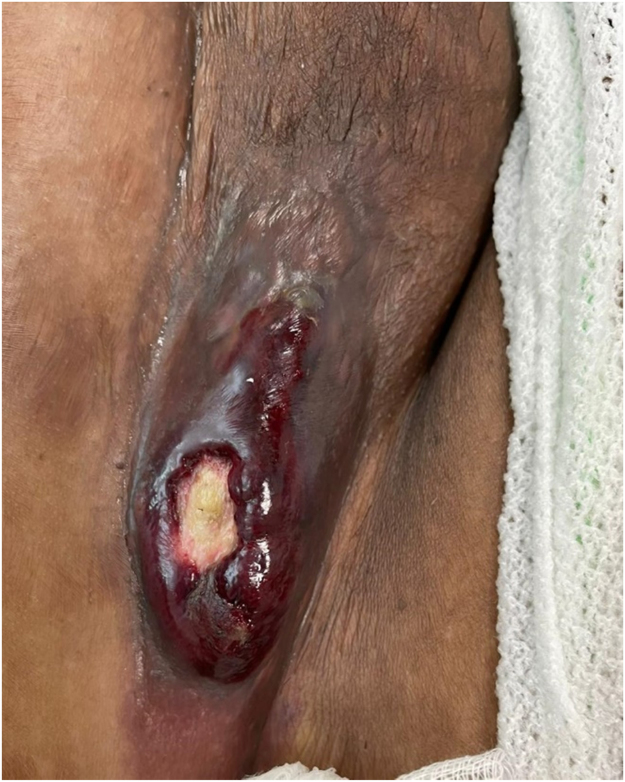
Skin lesion in the right inguinal area showing a necrotic hemorrhagic lesion typical of Ecthyma gangrenosum.

*Stenotrophomonas maltophilia*, an aerobic gram-negative bacillus, is emerging as an important opportunistic nosocomial infection rarely reported to cause ecthyma gangrenosum [1]. Importantly, *Stenotrophomonas maltophilia* is intrinsically resistant to numerous classes of antimicrobials, including beta-lactams, aminoglycosides, and carbapenems through various resistance mechanisms [2]. Trimethoprim-sulfamethoxazole is the empirical treatment of choice for *Stenotrophomonas maltophilia* infections despite it is comparable outcome with fluoroquinolones [3]. Early diagnosis and effective therapy of ecthyma gangrenosum are essential for improving prognosis and avoiding progression to life-threatening systemic infections in immunocompromised patients [4]. Therefore, *Stenotrophomonas maltophilia* should be considered in the list of culprit organisms when dealing with ecthyma gangrenosum in hematological malignancy.

## Funding

No funding was received towards the publication.

## Ethical approval

Ethics approval and permission was obtained to publish the case reports from the institutional review board which is in line with international standards,

## Consent

Written informed consent from the patient is available for review by the Editor-in-Chief of this journal upon request.

## CRediT authorship contribution statement

GA: Corresponding author, clinical management, data acquisition and manuscript writing. WG: contribute to data acquisition, manuscript preparation and final proof reading. MA and ASO supervised all the aspects and contributed to final manuscript editing.

## Conflict of interest

The authors declare that they have no competing interests.
